# HLA-DQB1 Allele Polymorphism Associated with Oral Submucous Fibrosis in Hunan, China

**DOI:** 10.1155/2024/8757860

**Published:** 2024-05-18

**Authors:** Yisi Tan, Yuting Huang, Linkai Guo, Linghang Zhou, Keke Zhu, Yuancong Li, Jin Tan

**Affiliations:** ^1^The College of Integrated Traditional Chinese and Western Medicine, Hunan University of Chinese Medicine, Changsha 410208, China; ^2^The First Clinical College of Chinese Medicine, Hunan University of Chinese Medicine, Changsha 410007, China; ^3^Department of Stomatology, The First Affiliated Hospital of Hunan University of Chinese Medicine, Changsha 410007, China

## Abstract

**Methods:**

44 OSF patients and 44 healthy volunteers were included in this study. To detect the expression frequency of HLA-DQB1 alleles in the two groups and analyze significant allelic subtypes and their relative risk, polymerase chain reaction (PCR) sequence-specific primers were used. Subsequently, based on the identification of differential genes, we compare the gene expression levels of OSF patients and healthy volunteers expressing differential genes by real-time quantitative PCR.

**Results:**

The expression frequency of the HLA-DQB1 ^*∗*^05 : 02 allele in the OSF group (36.4%) was significantly higher than in the controls (13.6%), and exposure to the HLA-DQB1 ^*∗*^05 : 02 allele was strongly related to OSF (OR (95% CI) = 3.619 (1.257,10.421), Wald *χ*^2^ = 5.681, *P*=0.017). However, there were no significant differences in the allele expression frequencies of DQB1 ^*∗*^02 : 01, DQB1 ^*∗*^03 : 03, DQB1 ^*∗*^05 : 01, DQB1 ^*∗*^05 : 03, DQB1 ^*∗*^06 : 02, DQB1 ^*∗*^06 : 03, and DQB1 ^*∗*^06 : 04 in the OSF group compared with the controls (all *P* > 0.05). Furthermore, the relative expression level of the HLA-DQB1 ^*∗*^05 : 02 allele in the OSF group (3.98 ± 3.50) was significantly higher than in controls (0.70 ± 0.41).

**Conclusions:**

There are differences in the HLA-DQB1 allele polymorphisms between the healthy population and patients with oral submucosal fibrosis. Preliminarily, it is suggested that the HLA-DQB1 ^*∗*^05 : 02 allele, which has a strong correlation with OSF and great differential expression between patients with OSF and controls, might be a susceptibility gene for OSF in Hunan.

## 1. Introduction

Oral submucous fibrosis (OSF) is a chronic and irreversible disorder of collagen metabolism, resulting in mucosal fibrosis and functional abnormalities. However, the pathophysiology of OSF remains incompletely understood, and numerous cases lack a clear cause [1, 2]. Previous studies found that the immunological environment of OSF altered considerably, leading to an imbalance in immune responses and tissue and cell fibrosis [2].

OSF is now a public health problem in many parts of the world, including the United Kingdom, South Africa, Sri Lanka, and Southeast Asian countries, as well as Hunan, Hainan, and Taiwan in our country. According to World Health Organization statistics, there are more than 5 million OSF patients globally [3, 4]. The malignant conversion rate of OSF is as high as 6%, but it varies widely between different ethnic groups and regions, such as 0.02 for Chinese, 0.08 for Indians, and 0.272 for Pakistanis [5]. Although chewing betel nuts is recognized as a major risk factor for OSF in Asia, epidemiological research indicates that chewing betel nuts is not the direct cause of OSF, and most betel nuts chewers do not show clinical symptoms or evidence of lesions [2, 6], and some individuals with severe OSF had only a brief history of betel nut chewing [7]. This inconsistent connection can only be interpreted by genetic predisposition [8]. Previous research found that genetic susceptibility has been linked to the development of fibrosis and carcinogenesis [9].

Human leukocyte antigen (HLA) genes are an essential component of the HLA system with high immunogenicity, playing a key role in the process of antigen processing and presentation, and their subarea HLA-DQ gene polymorphisms are associated with immunological function [10]. HLA genes are valuable genetic markers in genetic investigations due to the substantial genetic diversity and a strong linkage imbalance between different gene loci [11]. HLA typing and HLA gene frequency statistics are fundamental information for research on population genetics, individual identification, and the association between HLA and disease [12]. It should be noted that molecules encoded by HLA genes play an important determinant in immune response by presenting antigens to T cells and can be used as an important indicator to evaluate immune function [13]. HLA-DQB1 alleles are distributed mainly in immune cells, which can be used as indicators of the recognition of immune cells by one another, the induction of an immune response, and the identification of differences in disease susceptibility [14]. Furthermore, highly polymorphic HLA-DQB1 alleles have obvious racial characteristics within and between populations, and their gene distribution and expression vary greatly [14]. However, no studies have been conducted on their correlation with OSF susceptibility in most regions of China.

Therefore, the HLA-DQB1 allele was chosen as the main target of this study, and oral swab DNA extraction technology, polymerase chain reaction sequence-specific primers (PCR-SSP), and quantitative real-time PCR (qPCR) were applied to detect the expression frequency of the HLA-DQB1 alleles, analyze the meaningful subtypes of the alleles, and detect their expression levels. To study the susceptibility of OSF from the perspective of the HLA gene polymorphism for the first time in China. It will provide a new basis for the prevention and treatment of clinical OSF.

## 2. Patients and Methods

### 2.1. Ethics

According to the principles of the Declaration of Helsinki, the protocol for this case–control study was approved by the Ethics Committee of the First Affiliated Hospital of Hunan University of Chinese Medicine (approval no. HN-LL-YJSLW-2022-108). The informed consents were obtained from all participants.

### 2.2. Calculation of Sample Size

Power analysis was performed using *G* ^*∗*^Power 3.1.9.6 (Heinrich Heine University, North Rhine-Westphalia, Germany). The significance level on both sides is *α* = 0.05, *β* = 0.2, and *Power* = 1-*β* = 80%. After calculation, the total sample size was 88 (each group needs 44 participants), and the actual *Power* was 0.804. Taking into account the 15% shedding rate, 52 cases should be included in each group, for a total of 104 cases in the two groups.

### 2.3. Subjects

According to the inclusion and exclusion criteria and the calculated sample size, a total of 52 patients with OSF and 52 controls were recruited in this study. However, due to the poor quality and insufficient DNA concentration in some samples, the experimental data of 44 patients with OSF and 44 healthy volunteers were included in the final results of this study.

From June 2022 to December 2022, 52 patients with OSF from the First Affiliated Hospital of Hunan University of Chinese Medicine were recruited for this study. All of them were diagnosed with OSF. Other inclusion criteria included the absence of widespread, serious caries or extensive periodontal disease in the patient and other oral mucosal diseases. This study recruited 52 controls, whose age, sex, region, and ethnicity corresponded to the patients, without widespread, serious caries, extensive periodontal disease, or any oral mucosal diseases. The researchers meticulously documented the patients' age, sex, oral examination, history of betel chewing, smoking, and drinking, and took photos to record oral mucosal manifestations.

### 2.4. Sample Collection and DNA Extraction

Buccal swabs from all participants were collected according to the instructions of the Oral Swab Kit (Beijing Zoman Biotechnology Co., Ltd., Beijing, China) and stored in the oral cell preservation solution at room temperature. Genomic DNA extraction was performed according to the operating instructions of the Hi-Swab DNA Kit (Tiangen Biotechnology, Beijing, China). The purity and amount of DNA were determined by an ultraviolet (UV) spectrophotometer using the ratio of readings at 260 nm/280 nm. The OD260/OD280 ratio of sample DNA should be 1.7–1.9, and samples that were not in this range had been remeasured or DNA reextracted.

### 2.5. PCR Amplification and Electrophoretic Imaging

The total volume of PCR amplification was 20 *μ*L, including 0.5 *μ*g genomic DNA, 0.5 *μ*L Primer F, 0.5 *μ*L Primer R, and 10 *μ*L 2xTaq PCR MasterMix II (Tiangen Biotechnology, Beijing, China). The PCR cycle condition was 94°C for 3 min. This was followed by 35 cycles of 94°C for 30 s, 55–65°C (depending on the annealing temperature of each HLA-DQB1 allele primer calculated by the Tm Calculator) for 30 s, and 72°C for 1 min. Then 72°C for 5 min and the amplified samples were kept at 4°C. The PCR primer sequence of each HLA-DQB1 allele and GAPDH is listed in Table S1. The PCR products were then electrophoresed on a 2% agarose gel and visualized under UV illumination for gel documentation.

### 2.6. PCR Quality Control

The test samples were tested using the blind method; a negative control was established, and 10% of the samples were randomly selected for repeated experiments to test the reliability of the PCR results. Under the optimal reaction conditions for each primer, the positive sample should be amplified to its expected molecular weight, and there should be a complete and distinct highlight band in the electrophoretic result, without dispersion trailing and excessive nonspecific products, and the negative control should show no highlight band at all.

### 2.7. qPCR Amplification

The total volume of qPCR amplification was 40 *μ*L, including 100 ng genomic DNA, 1 *μ*L Primer F, 1 *μ*L Primer R, 1 *μ*L 50xROX Reference Dye 2 (Vazyme Biotech Co., Nanjing, China), and 20 L SYBR Green Master Mix (Vazyme Biotech Co., Nanjing, China). The PCR cycle condition was 95°C for 3 min. This was followed by 39 cycles of 95°C for 15 s, 55°C for 30 s, and 72°C for 30 s. Then 65°C for 5 s and 95°C for 5 s to collect the melting curve. Ct values were recorded after the reaction. The QPCR primer sequence and the GAPDH sequence are listed in Table S1.

## 3. Statistical Analysis

Statistical analysis was performed using SPSS 26.0 (IBM SPSS Inc., Chicago, IL, USA) and GraphPad Prism 10.0 (GraphPad Software, San Diego, California, USA). An independent sample *T* test was used for the comparison of age and gene expression levels between patients and controls. Differences in allele frequency, sex, and history of bad habits between the two groups were evaluated using the Pearson chi-square test or Yates continuity correction. The *P* value was corrected with the Bonferroni correction. The odds ratio (OR) and 95% confidence interval (CI) were calculated by a binary logistic regression equation. *P* < 0.05 was considered statistically significant.

## 4. Results

### 4.1. Demographic and Clinical Characteristics of the Study Participants

**Figure 1:**
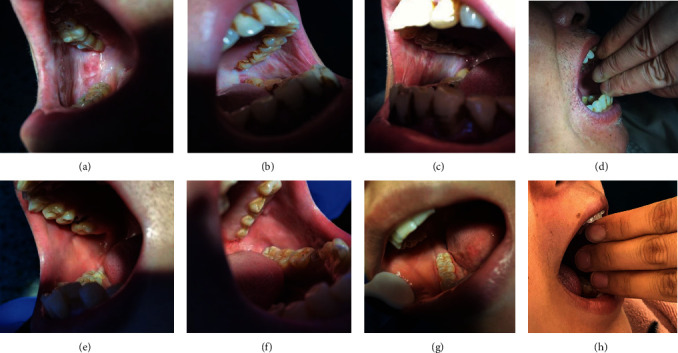
Comparison of oral mucosal manifestations in patients with oral submucous fibrosis (OSF) and controls. Note: Panels (a)–(d) are oral mucosal manifestations of OSF patients, and panels (e)–(h) are healthy oral mucosal.

This study included 44 OSF patients (42 men and 2 women) with a mean age of 41.86 ± 12.40 years and 44 controls (38 men and 6 women) with a mean age of 38.58 ± 12.89 years. There were no significant differences in age (*P*=0.016), sex (*P*=0.138), history of betel chewing (*P*=0.127), smoking (*P*=0.085) and drinking (*P*=0.672) between the OSF patients and controls (shown in Table 1). In patients with OSF, the oral mucosas were dry, blanched, and presented palpable fibrous bands, which can be seen mostly in the symmetry of the buccal. And the opening of the mouth was restricted. In comparison, the healthy oral mucosas were roseate, soft, smooth, and moist, and healthy subjects had no restriction on mouth opening (shown in Figure 1).

### 4.2. HLA-DQB1 Allele Frequencies in OSF Patients and Controls

**Figure 2:**
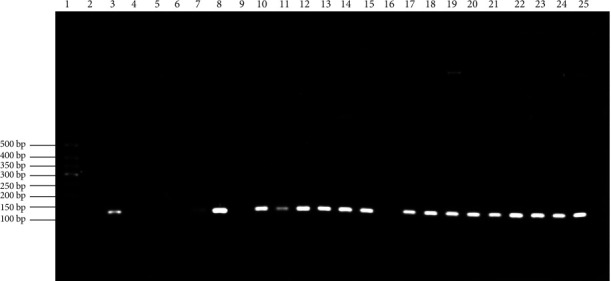
Amplified fragment of the specific human leukocyte antigen (HLA)-DQB1 ^*∗*^05 : 02 primer. Note: the target strip size was 127 bp. Lane 1 served as the marker and Lane 2 as the negative control. Lanes 3, 7, 8, 10–15, and 17–25 were HLA-DQB1 ^*∗*^05 : 02 allele-positive samples.

The DNA concentration and purity values of 20 randomly selected samples are shown in Table S2. All ratios werse within the range of 1.7–1.9, which met the standard. After genotyping 44 OSF patients and 44 healthy volunteers, the following 8 specific HLA-DQB1 alleles have been identified: DQB1 ^*∗*^02 : 01, DQB1 ^*∗*^03 : 03, DQB1 ^*∗*^05 : 01, DQB1 ^*∗*^05 : 02, DQB1 ^*∗*^05 : 03, DQB1 ^*∗*^06 : 02, DQB1 ^*∗*^06 : 03, and DQB1 ^*∗*^06 : 04. However, only DQB1 ^*∗*^05 : 02 had a statistically significant difference in expression frequency between the OSF group and controls, and its expression frequency in the OSF group was significantly higher than in the controls (36.4% vs. 13.6%, *P*=0.014; Table 2). The amplified fragment of the specific HLA-DQB1 ^*∗*^05 : 02 primer is shown in Figure 2. Meanwhile, there were no significant differences in the expression frequencies of the other 7 specific alleles between the two groups (all *P* > 0.05, Table 2). Furthermore, all samples from OSF patients and controls expressed DQB1 ^*∗*^03 : 01 and DQB1 ^*∗*^03 : 02 but did not express DQB1 ^*∗*^04 : 01, DQB1 ^*∗*^04 : 02 and DQB1 ^*∗*^06 : 01 (100%, 100%, 0.0%, 0.0%, 0.0%, Table 2). All of these alleles were nonspecific. Representative agarose electrophoretograms are shown in Figure S1.

### 4.3. Relative Risk of HLA-DQB1 ^*∗*^05 : 02 in the Development of OSF

The exposure of HLA-DQB1 ^*∗*^05 : 02 allele was significantly positively correlated with OSF (OR (95% CI) = 3.619 (1.257, 10.421), *P*=0.017, Table 3).

### 4.4. Gene Expression Levels of HLA-DQB1 ^*∗*^05 : 02 in Patients with OSF and Healthy Controls

**Figure 3:**
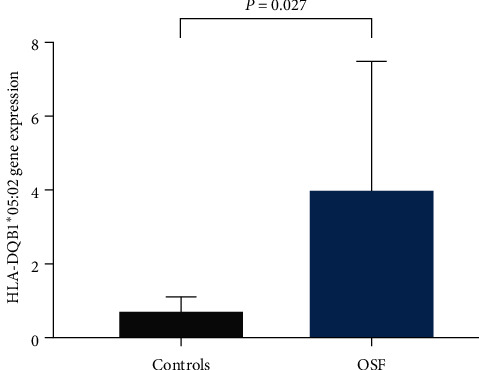
HLA-DQB1 ^*∗*^05 : 02 gene expression in patients with OSF and healthy controls.

The expression of the HLA-DQB1 ^*∗*^05 : 02 allele in the OSF group (3.98 ± 3.50) was significantly higher than in the controls (0.70 ± 0.41), and the difference was statistically significant (*P*=0.027, Figure 3).

## 5. Discussion

Oral submucosal fibrosis is a local mucosal disease and a precancerous state, so blood collection is uncommon in patients with OSF. As an invasive operation, blood collection can have negative physical and psychological effects on patients, which can be difficult for them to accept. Therefore, the key requirement for the successful completion of this study is the noninvasive DNA extraction of the study subjects. Previous studies have found the possibility of obtaining a sufficient amount of DNA for PCR by extracting DNA from oral swabs [15–18]. This is not only an economical, convenient, and effective method, but also a noninvasive sampling technique that patients find easy to consent to.

The development and occurrence of OSF are closely associated with immunological modulation. Oral mucosal inflammation is a major component in the pathophysiology of OSF, and the inflammatory site is where immune cytokines are produced, including interleukin, tumor necrosis factor, interferon, and transforming growth factor [19–21]. Researchers also found that people with OSF had considerably higher serum levels of immunoglobulin IgG and IgA [22–24], indicating the presence of an immunological response to OSF. In the course of clinical diagnosis and treatment, we discovered that there were other factors than local irritants that could cause oral submucosal fibrosis, such as betel chewing, smoking, drinking, and eating spicy foods. Some patients may not have any bad oral habits or environmental problems, suggesting that there may be a significant individual susceptibility to OSF. Susceptible populations may make the oral mucosa more vulnerable to lesions through chronic inflammation and the sustained release of inflammatory mediators [25].

Most of the time, a combination of environmental and genetic variables causes a disease rather than just one. The susceptibility determines how likely different people are to be affected in the same environment. On the other hand, high susceptibility is associated with a high risk of disease and a low risk of disease. However, gene expression plays a more critical role than environmental factors. In addition, the vast majority of cancers are also expressed in susceptibility, but in a different form. This susceptibility is present in the general population and in people who carry a particular susceptibility gene. Treatment of OSF focuses mainly on minimizing the symptoms of the patient and stopping the cancer from growing [26]. To reduce the occurrence of OSF, it is crucial to identify precancerous lesions at an early stage and excavate their etiology and pathogenesis.

Most HLA-Ⅱ genes are fundamental to the immune response and are associated with immunological function [14]. The HLA-DQB1 allele located in the HLA Class II gene region is mainly distributed in immune cells, which determines individual differences in disease susceptibility and is correlated with the incidence of OSF [8, 14, 27]. Purohit et al. [8] used PCR-SSP to determine the incidence of OSF in Indian patients and healthy subjects and discovered that the allelic expression frequencies of HLA-DRB1 ^*∗*^03 : 01 and HLA-DQB1 ^*∗*^05 : 03 were significantly increased in patients with OSF compared to healthy participants. Chen et al. [27] examined the HLA-A, -B and—C antigens, as well as the HLA-DRB1 and -DQB1 alleles of OSF patients using PCR-SSP DNA typing, and found that expression of the HLA gene in patients with OSF varied markedly. Furthermore, it is thought that certain specific HLA haplotypes may play a more important role in genetic susceptibility to OSF than individual HLA phenotypes. Kato-Kogoe et al. [28] also found that fibroblasts stimulated by HLA-DQ molecules promoted Th2 polarization in Th cell response and counteractivated collagen synthesis, suggesting a coordinating role for these cells in chronic inflammatory lesions with fibrosis. In addition, there were other genetic factors related to OSF in Hunan. Xu et al. [29] found that methylation of the CpG islands in the E-cadherin and cyclooxygenase 2 genes occurred more frequently in patients with OSF than in controls. Hu et al. [30] found that gene abnormalities in epithelial–mesenchymal transition could play an important role in the pathogenesis and malignant transformation of OSF. Low expression of LINC02147 also promoted OSF malignant progression [31]. Wang et al. [32] concluded that CircEPSTI1 might be an independent diagnostic and prognostic marker for patients with OSF. Cytochrome P450-related genes [33] played a role in the pathogenesis of OSF and lncRNA *ADAMTS9-AS2* [34] was expected to become a marker for the malignant transformation of OSF. These studies provided critical evidence to support the theory of genetic susceptibility to OSF.

In this study, after controlling for the demographic variables of the subjects, PCR-SSP was used to detect differences in the HLA-DQB1 gene polymorphism between OSF patients and healthy participants. We found that OSF patients had a considerably higher expression frequency of HLA-DQB1 ^*∗*^05 : 02 than controls, and the exposure of this allele was strongly positively correlated with OSF. Then quantitative detection by qPCR revealed that the gene expression levels of HLA-DQB1 ^*∗*^05 : 02 in OSF patients were significantly higher than those of healthy subjects. We preliminarily suggested that the HLA-DQB1 ^*∗*^05 : 02 allele may be a susceptibility gene for OSF, but no protective gene for OSF has been found in this study.

## 6. Conclusion

To summarize, there is a tight association between OSF and immune regulation. The HLA-DQB1 gene, as an important indicator reflecting immune function, has a certain relationship with OSF. Its subtype HLA-DQB1 ^*∗*^05 : 02 allele may be a susceptibility gene in the occurrence and development of OSF. These findings can provide valuable new clues for investigation into the mechanisms and development of new diagnoses and treatments for OSF.

## Figures and Tables

**Table 1:** Basic characteristics of the study participants.

Characteristic variables	OSF (*n* = 44)	Controls (*n* = 44)	*P*	*P*_*c*_
Age (years, mean ± SD)	41.86 ± 12.40	38.58 ± 12.89	0.145^a^	—
Sex (M/F)	42/2	38/6	0.138^b^	0.266
Betel chewer (*n*, %)	30 (75.00)	23 (52.27)	0.127^b^	0.191
Smoker (*n*, %)	29 (65.91)	20 (50.00)	0.085^b^	0.132
Drinker (*n*, %)	4 (9.09)	2 (5.00)	0.672^c^	0.672

OSF, oral submucous fibrosis. *Note*. *P*_*c*:_ corrected *P* value; ^a^independent samples *T*-test; ^b^Pearson chi-square test; ^c^corrected with Yates continuity correction.

**Table 2:** Distribution of human leukocyte antigen (HLA)-DQB1 alleles in oral submucous fibrosis (OSF) cases and controls.

Alleles	OSF (*n* = 44)		Controls (*n* = 44)		*χ* ^2^	*P*	*P*_*c*_
	Positive (*n*)	Frequency (%)	Positive (*n*)	Frequency (%)			
DQB1 ^*∗*^02 : 01	11	25.0	14	31.8	0.503	0.478^a^	0.636
DQB1 ^*∗*^03 : 01	44	100	44	100	—	—	—
DQB1 ^*∗*^03 : 02	44	100	44	100	—	—	—
DQB1 ^*∗*^03 : 03	9	20.5	11	25.0	0.259	0.611^a^	0.799
DQB1 ^*∗*^04 : 01	0	0.0	0	0.0	—	—	—
DQB1 ^*∗*^04 : 02	0	0.0	0	0.0	—	—	—
DQB1 ^*∗*^05 : 01	3	6.8	2	4.5	0.000	1.000^b^	1.000
DQB1 ^*∗*^05 : 02	16	36.4	6	13.6	6.061	**0.014** ^**a**^	**0.027**
DQB1 ^*∗*^05 : 03	26	59.1	31	70.5	1.245	0.265^a^	0.372
DQB1 ^*∗*^06 : 01	0	0.0	0	0.0	—	—	—
DQB1 ^*∗*^06 : 02	4	9.1	2	4.5	0.715	0.398^b^	0.672
DQB1 ^*∗*^06 : 03	2	4.5	0	0.0	0.512	0.474^b^	0.474
DQB1 ^*∗*^06 : 04	2	4.5	0	0.0	0.512	0.474^b^	0.474

*Note*: *P*_*c*:_ corrected *P* value; ^a^Pearson chi-square test; ^b^corrected with Yates continuity correction. The bold values are statistically significant.

**Table 3:** Binary logistic regression analysis of the relative risk of HLA-DQB1 ^*∗*^0502.

Variable	*β*	SE	Wald *χ*^2^	*P*-value	OR	95% CI
HLA-DQB1 ^*∗*^05 : 02						
Negative	—	—	—	—	1.000	—
Positive	1.286	0.540	5.681	0.017	3.619	1.257–10.421

CI, confidence interval; HLA, human leukocyte antigen; OR, odds ratio; SE, standard error.

## Data Availability

All relevant data are within the paper. All the raw data remain in the possession of the authors of the article.

## Additional Points

*Limitations of the Study*. This study is limited to the Han population in Hunan, and more sample sizes are needed to confirm whether this conclusion is correct and applicable to the Han population in other regions. This study only revealed the relationship between HLA-DQB1 and OSF susceptibility, and its specific mechanism is still unclear.

## Supplementary Materials

Supplementary Materials Table S1: the primer sequence of each HLA-DQB1 allele. Table S2: the DNA Purity and Concentration of Randomly Selected Participants. Figure S1: representative agarose electrophoretograms of nonspecific HLA-DQB1 alleles.
