# Supplementary material for: HLA-DQB1 Allele Polymorphism Associated with Oral Submucous Fibrosis in Hunan, China
Source: J Immunol Res. 2024 May 18;2024:8757860. doi: 10.1155/2024/8757860 (PMC11127765; doi:10.1155/2024/8757860)
Supplement: Supplementary Materials — Table S1: the primer sequence of each HLA-DQB1 allele. Table S2: the DNA Purity and Concentration of Randomly Selected Participants. Figure S1: representative agarose electrophoretograms of nonspecific HLA-DQB1 alleles. [file 8757860.f1.docx]

Supplementary Materials for

**HLA-DQB1 Allele Polymorphism Associated with Oral Submucous Fibrosis in Hunan, China**

Yisi Tan *et al*.

Correspondence author: Jin Tan, [tanjinhn@aliyun.com](mailto:tanjinhn@aliyun.com)

This file includes:

Table S1 to S2

Figure S1

TABLE S1: The primer sequence of each HLA-DQB1 allele.

| Gene | Primer | Sequence (5'-3') | PCR Products |
| --- | --- | --- | --- |
| DQB1*02:01 | Forward | TGC TAC TTC ACC AAC GGG AC | 188bp |
|  | Reverse | CTG TCC ACC GCC GCC CGT TT |  |
| DQB1*03:01 | Forward | TGC TAC TTC ACC AAC GGG AC | 111bp |
|  | Reverse | CGT CAC CGC CG GTA CAA CT |  |
| DQB1*03:02 | Forward | TGC TAC TTC ACC AAC GGG AC | 147bp |
|  | Reverse | GCT GTT CCA GTA CTC GGA GG |  |
| DQB1*03:03 | Forward | GGG ACC GAG CGC GTG CGT CT | 132bp |
|  | Reverse | GCT GTT CCA GTA CTC GGA GT |  |
| DQB1*04:01 | Forward | TTC ACC AAC GGG ACC GTA CT | 186bp |
|  | Reverse | GTA GTT GTG TCT GCA TAA GG |  |
| DQB1*04:02 | Forward | TGC TAC TTC ACC AAC GGG AC | 192bp |
|  | Reverse | GTA GTT GTG TCT GCA TAA GG |  |
| DQB1*05:01 | Forward | CGT GTA CCA GTT TAA GGT CC | 169bp |
|  | Reverse | GCT GTT CCA CTA CTC GGC AA |  |
| DQB1*05:02 | Forward | CGC CGC AGG GGC GGC CTA GC | 127bp |
|  | Reverse | CTC TGC AGG ATC CCG CGG TA |  |
| DQB1*05:03 | Forward | CGT GTA CCA GTT TAA GGT CC | 169bp |
|  | Reverse | CTG TTC CAG TAC TCG GCG TC |  |
| DQB1*06:01 | Forward | TGC TAC TTC ACC AAC GGG AC | 178bp |
|  | Reverse | CGT GCG TCT TGT GAC CAG CT |  |
| DQB1*06:02 | Forward | CGT GCG TCT TGT GAC CAG CT | 121bp |
|  | Reverse | GCT GTT CCA GTA CTC GGA AT |  |
| DQB1*06:03 | Forward | CGT GCG TCT TGT AAC CAT AC | 121bp |
|  | Reverse | GCT GTT CCA GTA CTC GGA AT |  |
| DQB1*06:04 | Forward | CGT GCG TCT TGT AAC CAT AC | 121bp |
|  | Reverse | GCT GTT CCA CTA CTC GGC AA |  |
| GAPDH | Forward | TCA AGA AGG TGG TGA AGC AGG | 115bp |
|  | Reverse | TCA AAG GTG GAG GAG TGG GT |  |

TABLE S2: The DNA purity and concentration of randomly selected participants.

| Samples | DNA Concentration (*μ*g/mL) | OD260/OD280 |
| --- | --- | --- |
| 06 | 44.04 | 1.876 |
| 12 | 46.23 | 1.832 |
| 20 | 56.41 | 1.828 |
| 21 | 41.43 | 1.847 |
| 25 | 38.84 | 1.864 |
| 28 | 44.13 | 1.829 |
| 32 | 89.07 | 1.853 |
| 33 | 40.38 | 1.899 |
| 37 | 61.87 | 1.822 |
| 40 | 57.48 | 1.886 |
| 44 | 74.56 | 1.795 |
| 52 | 69.43 | 1.855 |
| 63 | 95.81 | 1.886 |
| 66 | 74.25 | 1.757 |
| 77 | 65.53 | 1.746 |
| 80 | 52.61 | 1.777 |
| 82 | 61.74 | 1.830 |
| 85 | 47.11 | 1.876 |
| 87 | 60.60 | 1.859 |
| 88 | 116.00 | 1.872 |

**
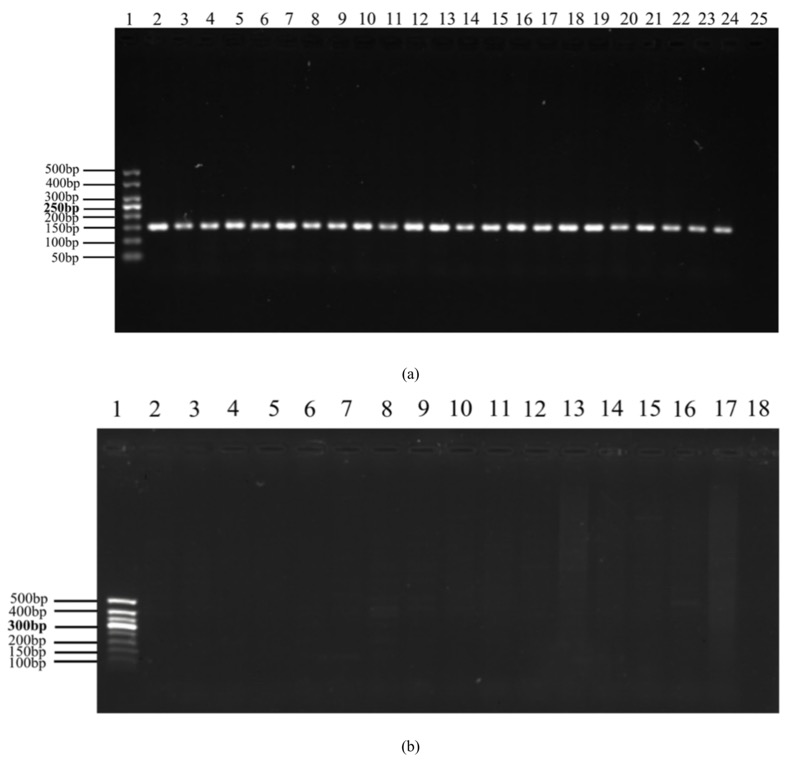
**

FIGURE S1: Representative agarose electrophoretograms of nonspecific HLA-DQB1 alleles. Note: (a) The target strip size was 147 bp. Lane 1 served as the Marker and Lane 25 as the negative control. Lanes 2-24 both were HLA-DQB1*03:02 allele-positive samples. (b) The target strip size was 178 bp. Lane 1 served as the Marker and Lane 18 as the negative control. Lanes 2-17 both were HLA-DQB1*06:01 allele-negative samples.
